# Women’s wellbeing as an empty declaration? A qualitative exploration of challenges in accessing termination of pregnancy due to fetal anomaly in Germany

**DOI:** 10.1186/s12910-025-01196-3

**Published:** 2025-03-21

**Authors:** Tamar Nov-Klaiman, Hilary Bowman-Smart, Ruth Horn

**Affiliations:** 1 Ethics of Medicine, Institute for Ethics and History of Health in Society, Faculty of Medicine, University of Augsburg, Universitätsstraße 2, Augsburg, 86159 Germany; 2 Australian Centre for Precision Health, University of South Australia, Adelaide, Australia; 3 Biomedical Ethics Research Group, Murdoch Children’s Research Institute, Melbourne, Australia; 4 Nuffield Department of Population Health, Ethox Centre, University of Oxford, Oxford, UK

**Keywords:** Abortion, Prenatal testing, Reproductive healthcare, Germany, Conscientious objection, Reproductive rights

## Abstract

**Background:**

The provision of prenatal testing through publicly funded healthcare systems, including non-invasive prenatal testing (NIPT), is frequently justified on the basis of supporting reproductive autonomy and informed choice. This includes decision-making around termination of pregnancy (TOP), including where it is due to a diagnosis of fetal anomaly (TOPFA). In Germany, TOP is regulated under the criminal code. However, it is exempt from punishment, if provided upon request from the woman up to 12 weeks after conception (14 weeks gestation) and following mandatory counselling. After this gestational stage, TOP may be provided where it is necessary to ensure the physical and mental wellbeing of the pregnant woman. However, there is a significant lack of clarity about how to interpret and apply this criterion. Fetal anomaly is often detected or confirmed after the time limit for TOP upon request has passed, which introduces uncertainty whether a fetal indication justifies legal access to TOP.

**Methods:**

This study explores attitudes towards TOP, experiences with decision-making and access, and the implications of the German legal and regulatory frameworks. It draws on a qualitative semi-structured interview study, conducted between 2021 and 2022. Participants were 20 German professionals who have experience or expertise regarding the provision of NIPT, as well as 7 women with experiences of pregnancy, reproductive decision-making and the offer of NIPT. Interviews were conducted in German, and then transcribed, translated, and analysed using thematic analysis.

**Results:**

Participants explored the importance of being able to access TOPFA; how the social positioning of TOP as a taboo procedure creates practical and psychosocial barriers to TOPFA access; the tension of who ultimately gets to make the decision about whether TOP can be provided; and how gestational time limits create emotional stress, frustrating informed decision-making and reproductive autonomy.

**Conclusions:**

Our findings highlight that where prenatal testing is provided in the absence of guaranteed access to TOP, women’s wellbeing becomes an empty declaration in German healthcare policy.

**Supplementary Information:**

The online version contains supplementary material available at 10.1186/s12910-025-01196-3.

## Background

In Germany, legislation and policy relating to reproduction and pregnancy is characterised by a unique ethical, legal and social context [1–3]. The official guiding principle to accessing both prenatal testing for fetal anomalies, as well as termination of pregnancy (TOP), is the wellbeing – mental or physical – of the pregnant woman (*die Schwangere*). However, while women’s wellbeing serves as the official criterion, a critical wider look at the legislative and policy frameworks raises questions as to whether they safeguard women’s wellbeing in practice. In this paper, we explore this tension and draw on a qualitative interview study with German professionals who have experience or expertise regarding the provision of non-invasive prenatal testing (NIPT), and women participants with experiences of pregnancy and reproductive decision-making.

Policies relating to prenatal testing are inextricably linked to access to TOP. The purpose of providing prenatal screening and diagnosis is often justified on the basis of supporting reproductive autonomy [4]. This can be broadly understood as the ability of someone to make and effect decisions about their own reproduction and pregnancy management, which may involve decision-making around TOP due to fetal anomaly (TOPFA). Therefore, providing prenatal testing while restricting access to TOP raises significant ethical issues, including the potential for psychosocial harms [5].

Under German law, the presence of a fetal condition does not in itself serve as an indication allowing access to TOP. Similarly, Germany does not offer population-wide prenatal screening targeted at specific genetic conditions such as trisomies 13, 18 and 21 (T13, T18, T21) [6]. Whereas other European countries, such as France and England, offer the combined-first trimester screening (CFTS) or NIPT to detect an increased chance of genetic conditions as part of their national prenatal screening programmes, Germany adopted a case-by-case approach [1, 7]. This means that NIPT is reimbursed ‘when the possibility of a trisomy burdens a woman so much that she wants it clarified’, as stated in German in the maternity guidelines published by the Federal Joint Committee [8].

German TOP legislation may be considered restrictive compared to other countries in Western Europe. TOP is criminalised under Sect. 218a of the Criminal Code (*Strafgesetzbuch*, StGB). However, it is permissible up until the 12th week after conception (i.e. 14 weeks gestation), with mandatory counselling and a 3-day waiting period (StGB Sect. 218a (1)). Afterwards it is permissible for ‘social-medical’ reasons at any gestation when deemed necessary ‘to avert a danger to the life or danger of a serious impairment of the physical or mental state of health of the pregnant women’ (StGB Sect. 218a (2)). While fetal anomaly as an explicit indication for TOP (or according to the German legal phrasing the ‘embryopathic indication’) was abolished in 1995 [9], it can still be used under the ‘social-medical’ indication to serve as an indirect reason for TOP (3). However, the 12-week limit for TOP without a required indication adds a strong sense of time pressure [10].

Questions around the ethics of selective reproduction, including procedures such as TOPFA, play a significant role in the German public discourse [11]. As in much of German policy and legislation, the desire to detach modern Germany from its eugenic past during the Nazi era was a key driver for the adoption of the *Embryo Protection Act*, the abolishment of the fetal indication from the law governing TOP, and the decision to fund NIPT in individual cases – rather than as a population-wide prenatal screening test [11–14].

In addition to legislative restrictions, a range of other considerations affect access to TOP, including TOPFA, in Germany. Not all clinics, nor all hospitals, perform TOP. Individual physicians – as well as institutions – can decide to refrain from providing the service on conscientious grounds. This applies to both early (within the first 12 weeks post conception period) and late TOPs [15]. Furthermore, until 2022, Sect. 219a of the German criminal code prohibited medical practitioners from advertising that they perform TOP along with providing information on the procedure and methods. This made it difficult for women to access information and to locate providing clinics [16–18], which undermined their ability to exercise reproductive autonomy and their health [19]. Restricted information serves to exacerbate inequities in access to TOP, since it disadvantages those with lower literacy. This affects patients' ability to access healthcare in general [20] and TOP-related care, in particular [21].

Now that the ban has been lifted, providers can advertise this service [22]. However, access to TOP remains difficult in Germany. Key challenges include the decisions of individual physicians and institutions to make use of their right to conscientious objection [15]; pressure from militant anti-abortion activism [23]; stigmatisation of the topic and insufficient education on TOP in medical school [24]. This, in turn, has ethical and social implications on physician–patient interaction as well as on a structural/procedural level, i.e., leading to insufficient providers and women struggling to access TOP, especially in rural areas and the southern and western areas of Germany [15, 17, 25, 26]. On top of the associated psychological distress [27] and the risk of women resorting to unsafe TOP [28], this hampers the basic ethical principles of health equity and reproductive autonomy [29]. The physical and mental health of women who fail to access a wanted TOP is threatened. Inaccessible TOP also challenges women’s ability to pursue education, establish a career, and achieve financial stability [29].

Although the German federal states are obligated by law to ensure sufficient numbers of abortion facilities, there are no regulations in place for the recording and evaluation of providers [26]. While data on the availability and accessibility of abortion care in Germany are lacking, reports of the Federal Statistical Office show a dramatic decrease in the number of TOP providers in Germany by 46.7% from 2003 to 2021 [26]. In Germany there are no guidelines to address the challenges posed by the possibility of physicians and institutions to refuse TOP provision due to conscientious objection. Unlike in Italy, physicians are not required to formally declare conscientious objection to the local healthcare authority [30]. As a result, monitoring of the phenomenon is more difficult, hampering the identification of regional gaps in service provision and the ability to ensure satisfactory supply. Without mandatory referral to providing colleagues and obligatory involvement of hospitals in TOP provision, as dictated for example in Portugal, the negative impacts of conscientious objection on women’s rights are exacerbated [15, 31, 32].

With the backdrop of the situation in Germany, it is vital to explore the views of those who are directly affected by it and operate within it. Through interviews with women participants and professionals, we explore experiences of, and attitudes towards, the provision of TOP and the implications of the current situation. This is particularly interesting within the German context where the legal and regulatory frameworks seem to convey contradictory messages. Both women seeking TOP and the professionals who treat them face these inconsistencies when the frameworks that allow prenatal testing for fetal anomalies and TOP on the premise that it can safeguard women’s wellbeing do not guarantee their access to TOP, even when it is the necessary procedure to ensure their wellbeing in practice.

## Methods

This work was conducted as part of a wider comparative empirical bioethics project examining the ethical issues associated with the introduction of NIPT into routine care in England, Germany and France.

The current paper examines the implications of the German legal and regulatory frameworks relating to TOP and TOPFA, drawing on interviews with professionals with relevant experience or expertise and women with experiences of pregnancy and prenatal testing.

### Recruitment

Professionals were recruited in Germany through established networks within prenatal genetics and policy, with subsequent snowball sampling. Women were recruited through flyers distributed in clinics of some of the professional participants and through invitations posted on organisations’ websites providing information about NIPT, such as the Down Syndrome Association. Semi-structured interviews (*n* = 27) were conducted in German online via Microsoft Teams by two experienced qualitative researchers, RH and HBS, between June 2021 and February 2022.

The inclusion criteria for the first group of participants were professionals (*n* = 20) who have been involved with, have experience with, or have other relevant knowledge regarding the provision of NIPT in Germany, including post-test counselling and return of results. Their primary roles, as described by the participants included: obstetrician/gynaecologist/fetal medicine specialist (*n* = 14); pregnancy or prenatal counsellor (*n* = 2); clinical geneticist (*n* = 1); and policy and advocacy (*n* = 3).

The second group of participants were women who had experience with pregnancy and the offer of NIPT within the German healthcare system. These participants (*n* = 7) were 30–50 years old. Six of them undertook NIPT in at least one of their pregnancies as well as various other tests, including ultrasound, chorionic villus sampling and amniocentesis. The other participant had only used ultrasound. At the time of the interview, five women were pregnant. Three women had terminated former pregnancies following abnormal test results. While recruitment of professionals continued until saturation was reached and no new themes emerged, recruitment of women was more difficult and continued until our internal project deadline.

### Data collection

Prior to the interviews, participants were provided with a participant information sheet detailing the purpose of the study, the funding body, and the institutional affiliation and role of the researchers. On the day of the interview, consent was obtained to conduct, record, and transcribe the interviews; to use anonymised quotes in scientific publications; to store de-identified transcripts, and to deposit these in the UK Data Archive. Consent was obtained verbally by reading the consent form out aloud and recording the participant’s indications of consent. A copy of the consent form signed by the interviewer was then emailed to the participant for their records.

Separate interview guides for women and professionals were developed for the study and were used to probe participants to express their perceptions and concerns—related to their role as either (formerly) pregnant person, or as a professional with relevant experience or expertise—concerning NIPT and pregnancy management (see supplementary files).

Once data were collected, the participant’s name was replaced by a unique participant number (pseudonymisation via a linkage list). The password protected list of participants names and contact details is accessible only to the interviewers, HBS and RH (PI), and will be kept for at least three years after publication or public dissemination and then will be destroyed.

Ethics approvals have been obtained from University of Oxford Central Research Ethics Committee (R64800/RE001). Data are available from the UK Data Archive for researchers who meet the criteria for access to confidential data.

### Data analysis

The interviews were transcribed verbatim, and identifying information about participants was removed prior to analysis. The interview transcripts were translated into English with the assistance of translation software. RH, a native German speaker, validated the accuracy of the translation for both coding and the use of selected quotes. Following a reflexive thematic analysis approach [33], the transcripts were coded and analysed using NVivo software by TNK, with cross-coding by RH and HBS. All researchers are experienced qualitative researchers. The reflexive thematic analysis followed the six phases as outlined by Braun et al. [33]. Furthermore, a collaborative iterative approach was used, with researchers positioned as cultural and linguistic insider/outside pairs to develop richer interpretations of the data [34]. We recognise the inherently subjective nature of the coding process and the potential of researcher subjectivity to affect coding reliability. The use of multiple coders, reflection on our own positionality, discussion of codes and assessment of the level of agreement between coders was purposefully used and served to overcome potential drawbacks [35]. This involved regular meetings between the researchers to discuss and review the construction of the meaning of the codes, with particular regard to their cultural and linguistic translatability. The core themes that were constructed through this process were ‘TOP as out of sight and out of mind’, ‘Whose decision is it?’ and ‘Racing against the clock to make a decision’.

## Results

Through the interviews, participants explored the ethical implications of the social discourse surrounding TOP and TOPFA, as well as the legal and regulatory frameworks regarding access to TOP in Germany. Participants described both the procedural aspects of TOPFA access, as well as their own attitudes and normative perspectives. This provided critical insight into some of the key challenges in accessing TOPFA in Germany.

For the purposes of identification, quotes are accompanied by participant number, as well as by participant group as either ‘Professional’ or ‘Woman’. The group labelled ‘Professional’ included the professionals with experience or expertise in reproductive policy or healthcare provision, and identifiers also include their gender. The group labelled ‘Woman’ includes the women we interviewed with a focus on their experiences related to pregnancy, reproductive decision-making, and NIPT; their identifiers also include a general description of their profession. Quotes with an asterisk (*) were previously reported in our preceding manuscript [3], but were also captured by the themes in the present analysis and are reported here.

### TOP as out of sight and out of mind

An overarching finding, emerging from both groups of participants, was how TOP is more generally positioned, in both social discourse and legislation, as a procedure that is morally problematic and subject to taboos. As a consequence, TOP as a procedure is – as we describe here – kept out of sight and out of mind. TOP in the abstract might be foregrounded in ethical and social debate, but the actual provision of TOP as a medical procedure becomes hidden and unspoken. This social positioning of TOP leads to substantial material and practical difficulties in accessing TOPFA, especially at more advanced gestational ages, the timeframe in which fetal anomaly is usually diagnosed.

One way that TOP is kept out of sight is through difficulties in finding physicians or facilities who can provide it. One major reason mentioned was the insufficient number of providers. This was suggested to be primarily the result of physicians making use of their right to conscientious objection. A gynaecologist who is also working in a family counselling centre explained that this is the case not only with individual physicians, but at the level of entire facilities or clinics:

‘The vast majority of clinics back out and don't perform that [TOP], because there is the option of not performing abortions for conscientious reasons.’

[Participant #11, Professional, Gynaecologist, female]

Indeed, severe shortages of providers were repeatedly described, for example by another gynaecologist and psychotherapist who is working as a counsellor for prenatal diagnosis:

‘The supply situation [of TOP] is becoming increasingly difficult. We see that all over Germany. So, in the last ten years, I think the number of physicians performing TOPs has gone down by 40%. […] That's being hotly debated all over Germany, including in the association. And here in [state in the Southern part of Germany] we don't have coverage throughout. We have regions, counties, in which there are no physicians left who perform TOPs.’

[Professional, Gynaecologist and psychotherapist, female]

Such reports go hand in hand with women’s accounts of their own experiences as well as those of their acquaintances. One of the participants, a mother of a child with a disability, whose second pregnancy was terminated following a prenatal diagnosis of Down syndrome, described her experience seeking a TOPFA:

‘It was last year [2021] when we terminated... The entire team worked to ensure that we got an appointment as quickly as possible because the situation was just so terrible for everyone. It was really great how hard everyone tried, but… how difficult it was to get an appointment. Because hardly any clinics do it anymore.’

[Participant #13, Woman, Psychologist]

The quotes above uncover the profound scale and impact of the shortage of physicians and facilities offering TOP. Following a constant decline over the years, the situation now is such that significant areas in Germany lack sufficient access to TOP services. This means that while women may have access to prenatal testing and diagnostics to confirm a fetal anomaly, if they do decide to seek a TOPFA, they will likely struggle to get it.

Another reason for women’s difficulty to access TOP was the former ban on clinics from publicly stating that they provided TOP. As a result, women were not only facing an insufficient supply, but also a ‘hidden’ one. As one participant who terminated her first pregnancy following the detection of a fetal anomaly explained:

‘They [the clinics] are not allowed to advertise them [TOP procedures]. That means that regular people cannot find those who are willing to carry it out. I also dealt with the topic and looked where I can best have a TOP. I found nothing. My prenatal diagnostician then had to tell me about the clinic where it was carried out.’

[Participant #20, Woman, Statistician]

This participant, as well as others who referred to the ban and its implications, expressed how such under-the-radar situation turned locating clinics into an even greater struggle. This demonstrates how inconsistencies between different parts of the policy and legislative framework governing TOP – namely, permitting and funding TOP while prohibiting information about it – impact women in practice and translate into increased stress.

The TOP advertisement ban has since been removed, with the intention to eliminate the technical obstacle to locating providers and to information on the procedure [22].

Participants described a social climate disapproving of TOPFA, which becomes another obstacle women face. A judgmental outlook on TOP is not confined to the medical sphere in which women are treated. Rather, participants suggest that this type of attitude towards TOP are commonplace more broadly in society. This includes a public debate that is often critical toward testing and subsequent TOPFA, as this physician described:

‘In many contexts it is very much a taboo, the topic of prenatal diagnostics and how to deal with it [TOPFA]. […] The debate is very moralising and that is certainly not helpful.’

[Participant #15, Professional, Physician, female]

Women participants also referred to a broader social environment that positions TOP as ‘taboo’. Some who had themselves undergone TOPFA described how they resisted this taboo, or wished it were not the case. One participant, who terminated her pregnancy after a fetal diagnosis of Down syndrome, framed TOP as a subject that is not taboo *for her* (while indicating it is for others), and that she freely speaks about her experience with TOP. For this participant, TOP as taboo was seen at a more granular level in terms of individual views and attitudes. Another participant also focused on the positioning of TOP as taboo, but emphasised the societal level of the taboo. She focused on the nature of reproductive decision-making as individual and personal as a reasoning for why TOP should not be taboo.

‘For me it’s not a taboo subject in any way. […] I had to tell everyone that it didn’t work out and why. But that’s okay with me, I’m now behind it, even if some still think it’s crazy that you can have an abortion for that.’

[Participant #19, Woman, Sales manager]

I wish that abortion was not considered a taboo. We live in a free society, and it is permitted by law. So why make it such a taboo? It is for the individual to make this ethical decision of what suits them and is justifiable.

[Participant #20, Woman, Statistician]^*^[3]

Participants generally focused on the need for a change in societal outlook on TOP, and argued that ‘accept[ing] their decision without judgment’ (Participant #23, Woman, HR Manager) was the best way to support women in their reproductive path.

### Whose decision is it, really?

A strong signal from interviews with both groups of participants was the significance and critical necessity of access to TOPFA. In the face of criticism towards NIPT and selective reproduction in the German social and political discourse, participants highlighted access to TOPFA as important for reproductive autonomy while emphasising the personal nature of reproductive decision-making.

Some participants framed TOPFA access as a matter of individual freedom and choice. One participant argued that while there are loud groups opposing the use of NIPT and selective TOPs – driven by arguments of social inclusion and religiosity – these are small groups engaged in ‘Crusades’, whereas the ‘silent majority’ is supportive.

‘There is a large silent majority that approves of this test [NIPT] and that would ultimately support TOP due to fetal anomaly. That must be clear. So, we don't need Crusades here, we need a freedom that allows everyone to do the right thing in their world.’

[Participant #2, Professional, Policy-maker, male]

Participants who had experiences of pregnancy – such as the following participant who undertook NIPT but had a result indicating a low probability of an aneuploidy – emphasised how personal circumstances in one’s life could lead to the decision to terminate following the diagnosis of fetal anomaly. She described the importance of each person being able to ‘find the right way’ in their own individual circumstances and how without access to procedures such as TOPFA, parents may ‘break’.

‘There was the question: ‘What if my own result indicated a trisomy?’ I hope I would have kept the child, but I’m not 100% sure. […] I don’t think that disability makes people unworthy of life. But as I said, there are many circumstances that play a role. You have to be able to do it as a parent. If the parents break because of it, that’s not good for the child either, so you have to find the right way.’

[Participant #23, Woman, HR manager]

These accounts convey the significance of making TOPFA accessible to women. However, as the findings indicate, it is not straightforward for women in Germany to act on their reproductive decisions.

When discussing the pathway to TOPFA access and decision-making, a theme that emerged was the ambiguity and tension of who really gets to make the decision about a woman’s access to the procedure. While the importance of autonomy and choice in reproductive healthcare may be emphasised in policy, participants described how the decision to provide TOP after detection of fetal anomaly ultimately depends on either individual healthcare professionals or legal and regulatory restrictions.

For example, participants described how women must obtain their treating physicians’ approval to be eligible for TOP. This is perceived as an obstacle in their decisional path. This obstacle is twofold: procedural and emotional. With the pregnant woman’s physical or mental state of health as the only criterion for TOP under the medical indication (from week 12), such approval is solely dependent on the physician’s own evaluation.

‘A medical indication must be issued by a physician. Ultimately it is not the couple who decides about it [TOP]. At best that’s consensual. Some couples think that if something is found, they can decide. That’s not true. Usually there is a conversation, and you and the physician then come to a common stance on how to proceed, but the couple doesn’t decide. Sometime there is controversy.’

[Participant #16, Professional, Gynaecologist and psychotherapist, female]^*^[3]

The road to receiving such an approval is often complex. Even in cases where the approval for the indication is issued, women are sometimes confronted with discouraging healthcare staff. One participant who terminated her pregnancy following the detection of a chromosomal condition was adamant to terminate her pregnancy. She recounts, however, how multiple physicians tried to deter her from opting for TOP and how difficult it was to obtain the certificate of approval; she states that the approving physician referenced that she ‘would be a single parent’ as part of his reasoning.

‘After the final amniocentesis, it was actually clear to me [the wish to terminate]. Only the physicians tried to convince me that I would not terminate. […] The physician in the diagnostics centre said that he was very reluctant to give out this letter for an abortion and that I should be extremely sure about it. And then he sent me to pregnancy conflict counselling and said I also had to talk to the paediatrician who specialises in children like this. […] I then had a final conversation with him, where he acknowledged that I had spoken to the paediatrician and had this [pregnancy conflict] consultation, and that I actually did everything that was important to him. [He said that] since I would be a single parent it is okay for him to issue this certificate and then referred me to the clinic.’

[Participant #20, Woman, Statistician]

After describing her odyssey to access a desired TOP, Participant #20 concluded by saying that the process made her feel ‘very alone’. During such a vulnerable time and in the face of discouragement from healthcare staff, it is emotionally demanding on the women to insist and pursue their desire to terminate.

Participants raised concerns about women losing autonomy in decision-making over their pregnancy management. Healthcare professionals must find a reason to provide TOP that aligns with the wording of the legislation, rather than just the woman’s decisions or preferences. In the words of the following healthcare professional:

‘You have to tell the woman that she no longer has any right to make her own decisions. An indication must be given, which must not be eugenic, but a medical indication that relates exclusively to women’s health. Someone needs to make this indication, that the woman’s health is so affected by this pregnancy that there is no other option than to have a TOP. And that must be a different physician than the one who performs the termination. She then needs a clinic that implements it, that actually performs the termination. The woman can no longer decide.’

[Participant #11, Professional, Gynaecologist, female]

Women with experiences of pregnancy interviewed for this study further underscored reproductive autonomy and articulated how important is access to information on testing – and through testing – as well as potential subsequent services as TOP. They emphasised the personal nature of reproductive decision making and stressed that it should be left for women to make, as they ‘must live with the consequences’ for their decisions. As one woman who undertook NIPT during her current pregnancy articulated:

‘I think that if I decide to test whether I’m expecting a child with a disability and then possibly terminate – that’s a very, very subjective decision, because I personally must live with the consequences. […] Everyone must decide for themselves which tests to do or not to do.’

[Participant #25, Woman, Teacher]

Criticism was raised over the situation in Germany, where access to information and services is unequally distributed, thereby contributing to potential inequities in access to reproductive healthcare. One participant highlighted her concerns around injustice and inequities:

‘Injustices occur, because access to medical knowledge and knowledge about preventive services is distributed very unequally in Germany.’

[Participant #24, Woman, Academic]

Several participants claimed that the legislation and policies related to TOP in Germany encourage ‘abortion tourism’. They suggested that some women seek TOP abroad due to the barriers of accessing it in Germany. As one participant, a gynaecologist, described:

‘Of course we are promoting a kind of tourism. [People decide that] If it doesn’t work here, we’ll get it somewhere else. That was the case with preimplantation diagnostics, that’s the case with reproductive medicine, whether it’s surrogacy, egg donation or whatever. I think that causes a lot of suffering.’

[Participant #16, Professional, Gynaecologist, female]

The current framework for TOP provision in Germany can take the decision out of the hands of those seeking TOPFA, whether that is through a physician directly denying the procedure, or other inequities in access. However, the importance of women being able to exercise their reproductive autonomy and make the ultimate decision about TOPFA was stressed by participants. The interviews indicate that where the decision about reproductive healthcare is removed from women’s hands, they may seek to reclaim it, such as by travelling to another jurisdiction.

### Racing against the clock to make a decision

Participants felt that people seeking TOP in complex situations, such as where there is a diagnosis of fetal anomaly, are often forced to make an important or weighty decision under significant time pressures. These may affect whether someone is able to access a particular type of TOP procedure, or able to access TOP at all.

References were made to two changes in TOP management that occur around the same point of gestation in the pregnancy. One is the end of the period during which women can terminate upon request without providing a reason, although pregnancy-conflict counselling is required (up to week 12 post-conception). This is followed by the medical indication period which mandates the approval of a medical professional. The other change is the limit of the period during which TOP is offered surgically in Germany, e.g., through vacuum aspiration (until 12 weeks), and after which TOP is performed through medically induced labour which may be associated with a larger emotional and physical impact on the woman. One physician specialising in obstetrics and perinatal medicine referred to the time limit for TOPs upon request and explained that when women rush to terminate following a positive NIPT result without a diagnostic test, it remains unverified whether the result was true or false positive:

‘A positive NIPT is not an indication for TOP. This is a big problem if you do NIPT in the 9th week, because there is a regulation in Germany that allows women to have an abortion up to the 12th week of pregnancy as part of the time limit regulation. If I do NIPT on a 25-year-old in the 9th week and it comes back positive and she terminates in the 11th week, nobody ever knows whether the child had trisomy 21 or not.’

[Participant #21, Professional, Prenatal medicine specialist, male]

A therapist working in a counselling centre on pregnancy and prenatal diagnosis addressed how increasing gestation affects the particular method of TOP that might be offered, and expressed similar concerns:

‘Time pressure is inherent to the decision anyway, but there is an additional external pressure, namely this difference in the termination method. Our fear is that this will lead to women no longer waiting for the diagnosis after a positive NIPT so that they can still have vacuum within the deadline, but at the risk of the child having nothing at all.’

[Participant #7, Professional, Therapist and prenatal counsellor, female]

These professionals’ concerns are supported by the accounts of the following participant who, by the time the diagnosis confirmed a fetal anomaly, could only terminate through induced labour which caused her great distress:

‘I said to myself for future pregnancy, that as soon as there is a suspicion, I’d rather have an [early] abortion, because I don’t want to go the way I had to go afterwards [induced labour]. That was the horror for me.’

[Participant #20, Woman, Statistician]

These quotes demonstrate the emotional stress that can be caused by the short timeframes related to both factors – the period allowing TOP upon request, and the period allowing surgical TOP. Women may feel the need to reach a decision quickly before the time window is shut. Moreover, as several participants highlighted, this may increase the risk of women deciding to terminate based on inconclusive results from screening tests like NIPT – which has considerably high false positive rates for some conditions – in order to be eligible for TOP before 12 weeks. Women might, therefore, be driven to TOP without confirmatory diagnosis, potentially terminating unaffected pregnancies when they would otherwise not have chosen to do so.

It was argued that other neighbouring countries, such as the Netherlands, offer easier access to TOP in terms of locating providers, a significantly longer period during which TOP can be performed without indication and longer time frames for surgical TOP. A patient representative and a mother of a child with disability herself addressed the significance of the availability of surgical TOP in the second trimester. Of note, despite her claim that vacuum aspiration is in use in the Netherlands beyond 12 weeks, there are in fact other forms of surgical TOP, e.g., dilatation and evacuation, during this period.

‘I don’t think it is good that you can terminate pregnancy with the suction method only up to the 12th week. So, the time pressure is enormous. […] I find that really dramatic. […] In order for women to be able to make a self-determined decision, many more possibilities must be created, both in terms of safe TOP and life with a special child. It would be wonderful if, like in the Netherlands, it were possible in Germany to be offered a suction method nationwide up to the 22nd week if there is a medical indication.’

[Participant #12, Professional, Disability advocate, female]

The quotes above reflect the need for longer timeframes to allow for informed and free decision-making during pregnancy. They underscore the importance of allowing women to verify prenatal diagnoses and reach a decision based on conclusive results rather than being coerced by strict timelines or concerns about the method used at particular gestations. Less time pressure allows for more deliberation and facilitates self-determined decision-making.

## Discussion

This paper sheds light on the ethical, social and psychological implications of the current offer of and access to TOP in Germany, particularly following the detection of fetal anomaly. It provides further insight into how professionals in the field and women seeking reproductive healthcare are impacted by the situation and perceive it. This is of special interest, since the German legal and regulatory frameworks that govern prenatal testing and TOP, and their underlying values, are conflictual in practice [3].

### Regulatory ambiguities, inconsistencies, and recent progress

Our results highlight key challenges in the journey to access TOPFA. After a prenatal diagnosis, a pregnant woman must put forth her case to get the approval; then she must search for a physician who will perform the TOP; and meanwhile, she must herself make a weighty decision under significant time pressure. For many years, German legislation and regulation have been presenting an inconsistent approach to TOP and sending contradictory messages. For example, the state is required to protect ‘unborn life’, and TOP is regulated by the criminal code; however, the state is also obligated to fund the infrastructure and facilities necessary to provide TOP [16]. Legislation such as the Pregnancy Conflict Counselling Act explicitly states that the counselling serves to protect unborn life, but the counselling must also be non-directive and the choice must ultimately be the pregnant woman’s [1].

Moreover, on the one hand, policy and legislation put much focus on supporting women’s physical and mental wellbeing and emphasise the importance of free and informed choice. Whilst this attitude is reflected in the interviews, it became apparent that legislative and regulatory barriers to accessing TOP are in fact compromising women’s wellbeing and frustrating their choices. The former ban on physicians and clinics from advertising the provision of TOP services and giving information on the procedure and pathways is perhaps the most conspicuous example of such inconsistencies. The recent reform of the law, allowing physicians to declare TOP provision and give information about the procedure [22], is therefore an important step in addressing inconsistencies in the law (permitting TOP under certain circumstances and requiring the state to ensure sufficient supply of providers, while simultaneously prohibiting them from advertising themselves and providing information about TOP). It reduces access barriers for women trying to locate providers and information, while alleviating the fear of practicing professionals from ramifications. Other encouraging steps have included a recent report by a government commission recommending the decriminalisation of first trimester TOP, and the removal of mandatory counselling requirements [36].

However, this does not solve the gap between the law’s focus on women’s wellbeing and their ability to realise their wishes and needs in practice. Lifting the ban on TOP advertisement did not address another difficulty, namely the interaction of women with their treating physician, required to approve eligibility for TOP beyond the 12 weeks limit. The absence of fetal anomaly as a clear indication for terminations leaves much room for physicians to act according to their own views, even when it is against women’s wishes [25]. Indeed, some of our participants described different ways in which women’s autonomy is compromised. They explained that without an official fetal indication, professionals must issue a medical indication that relates exclusively to women’s health and are thereby left with less room to manoeuvre and accommodate women’s wishes. Physicians that are a priori against terminations can more easily delay and hamper women’s path to TOP, as has been described by our participants and in other studies [15].

The absence of a fetal indication from the law regulating TOP does not just negatively impact pregnant women. It also leads to uncertainty among physicians and lawyers [37] and potential conflicts with law enforcement authorities [38], consequently discouraging physicians from assisting women in terminating pregnancies in cases of fetal anomaly [25]. When healthcare professionals operate within a framework that suffers from lack of clarity in the criteria for TOP, this can negatively impact service delivery and has the potential to harm their patients. Moreover, scholars have questioned whether the ability to assess the expected impact of a pregnancy on a woman’s physical and mental health, as currently required in the medical indication, is within the scope of physicians’ medical knowledge, and have called for better regulation and change in the framework [39].

### Conscientious objection and conflicting messages in prenatal care

In this study, both participants who were healthcare professionals as well as women who had sought TOPFA described the difficulties in finding a TOP provider, despite the obligations of the federal states to ensure sufficient supply. Physicians and facilities who provide TOP were described as both scarce and elusive, with participants mentioning the impact of conscientious objection.

Conscientious objection has been highlighted as one of the main drivers of the severe insufficient supply of TOP services throughout Germany [15, 24, 40]. This affects access to TOP at all gestations, including first trimester TOPs made upon request. However, where TOP is sought after a prenatal diagnosis, conscientious objection can interact with and compound stigma relating to selective reproduction. The case of TOPFA emphasises, therefore, another overarching inconsistency – the one between the regulations relating to different stages of pregnancy management, namely prenatal testing and TOP. Where a healthcare professional is willing to provide prenatal testing, but refuses to provide TOP after diagnosis, this creates conflicting messages in the prenatal care setting. As Krawutschke et al. explain: ‘It might seem cynical from the perspective of a pregnant woman, if the same physician who recommended prenatal testing to her, retreat to conscientious objection when it comes to abortion as a consequence’ [15].

Therefore, conscientious objection and the lack of its regulation can function to undermine reproductive autonomy and women’s human rights [41, 42]. It has ethical implications since it impedes equal access to TOP, both geographically and economically, and negatively impacts women’s physical and emotional wellbeing [15, 30, 43, 44]. Conscientious objection has notable social ramifications as it strengthens the stigma associated with TOP and it unequally positions women according to their socio-economic status and place of residence [42]. Both groups of participants interviewed for this study referred to the severe lack of TOP providers causing additional emotional stress, time pressure, or financial burden where women are forced to travel long distances, including to neighbouring countries [18]. Inaccessible TOP becomes a matter of health inequity and social injustice [5, 10, 28].

In line with previous works and reports [17, 18], professional participants in our study pointed to insufficient and uneven coverage of TOP throughout Germany, with some areas containing no providers at all. We therefore recognise that even if the law governing TOP were such that made it more straightforward, a significant bottleneck would remain – that of insufficient offer of TOP by providing clinics. This requires a separate solution to go in parallel with changes needed in the law or policy governing TOP, to ensure supply that guarantees equal access to the service [31, 43, 45]. Measures could include improved monitoring of the phenomenon through obligatory declaration of conscientious objection and updated registries as well as mandatory referral to providing colleagues and obligatory involvement of hospitals in TOP provision [15, 30–32].

Another critical factor is the taboo and social stigma intensified by the criminalisation of TOP, and the association of TOPFA and selective reproduction with Nazi eugenics. The nature of the social discourse on TOP affects women’s sense of legitimacy to pursue it, as well as that of healthcare professionals to provide it [3, 24, 46, 47]. While studies on attitudes toward TOP in Germany are scarce, some have demonstrated increase in negative attitudes towards TOP during the two decades following Germany’s reunification, pointing to the intertwined relationship between policy/legislation and social stigma, which ultimately impacts women’s rights and health [46]. Our findings follow this, with our participants raising significant concerns about the positioning of TOP as ‘taboo’ in social and political discourse and its impact on their experiences. In such a social climate – and without proper regulation – physicians with a negative view on TOP can easily make use of conscientious objection as a tool to avoid offering it. This ultimately translates into severe lack of TOP services [24]. Inaccessible TOP arguably makes this topic more of a taboo, which in turn further discourages professionals from providing it, in a cycle that increases the distress experienced by women facing the decision to terminate. While the intention behind the changes made in TOP-related legislation in Germany (e.g., the abolishment of the embryopathic indication) was to tackle value judgements about life with disability [37], there is no evidence that the current TOP legislation in Germany strengthens disability rights compared to other European welfare states [48]. Rather, it has been argued that the entanglement of reproductive and disability rights obscures open debates about obstacles to an inclusive society [2].

### The risks of hurried decisions under restrictive time limits

The challenges in finding a provider and navigating conflicting messages are compounded by the strict timeframe placed on the decision-making processes. Participants in our study raised concerns about the possibility of women being forced to make hurried decisions and/or seeking TOP without getting confirmatory diagnosis. In addition to the short timeframe allowing for TOP upon request (within 12 weeks post conception), the incentive to seek access to TOP abroad may arguably be even stronger due to the restrictions on methods used in Germany. TOP with vacuum aspiration is performed only until 12 weeks, afterwards labour is induced medically [49]. Other countries like the Netherlands or the US offer surgical TOP beyond 12 weeks [50, 51], hence, responding to many women’s preferences of choosing dilation and evacuation over labour induction to avoid the birth process and the method being faster and with less personal contact with the fetus and a lesser physical impact [52].

Therefore, as some participants highlighted, this arrangement carries the potential to create pressure on women to make decisions early in gestation, when vacuum aspiration is still an option. This is particularly relevant in the case of NIPT, which can be done early in pregnancy and provide results within the period when vacuum aspiration is possible. The limited timeframe during which surgical terminations are available in Germany adds to the time pressure women experience as a result of the limited period for TOP upon request, beyond which the approval of medical professionals is necessary for TOP. Some of our participants raised concerns that women who hasten for early termination might opt for TOP following a positive NIPT result without confirmatory diagnostic testing, at the risk of terminating unaffected pregnancies. Clinicians elsewhere have flagged this, with some suggesting an association between the increasing number of early TOPs in 2023 with the broad availability of NIPT leading to pregnancy decisions based on unconfirmed results [53]. Relying on uncertain screening results, however, may undermine reproductive autonomy, which is based on informed decisions, and is essential for reproductive health [19].

While Germany presents a concerned attitude toward TOP for fetal anomaly and its practice, in other places, e.g., Denmark, there are attempts to frame such TOP as an act of love and responsibility by adopting practices of care through clinical guidelines and patient-caregiver interaction. This serves both couples and healthcare staff in coping with the moral burden associated with selective terminations and repositioning them as permissible acts [54].

The implications of inaccessible TOP do not end at the time of birth, but rather are long lasting. Those who eventually cannot achieve a wanted termination are disproportionally affected for years to come. Studies showed that compared to women who achieved termination, women denied of a wanted TOP were more prone to poverty years after birth [55] and that women receiving wanted terminations had similar or better mental health outcomes than those denied of them [56]. Since the German legal and regulatory frameworks of pregnancy management – both for prenatal testing and TOP – highlight women’s physical and mental wellbeing on the declarative level, such findings must be considered when examining and revising these frameworks. In line with this, some scholars call for frameworks that allow TOP on request of the pregnant woman rather than making TOP available based on grounds, with the claim that this is warranted from a rights-based perspective. Such an approach would facilitate women’s reproductive autonomy and their assessment of what is right for them [28].

### Study limitations

The study draws on interviews with a small group of participants. In particular, the number of women that we interviewed to focus on their experiences of pregnancy and reproductive decision-making was much smaller than the cohort of professionals. The difficulty to recruit women for the study could be related to two aspects. First, it is likely to be emotionally difficult for women to talk about their experience with NIPT, in particular in the event of a positive finding, during or shortly after an affected pregnancy. Since we recruited through clinics or websites providing pregnancy or NIPT information, most women who read about our study were timewise close to pregnancy. Second, the stigma surrounding the topic and the social positioning of prenatal testing and TOP as taboo, as indicated by our participants, could have played a significant role in deterring women from sharing their experiences. The small cohort may have resulted in a limited range of viewpoints. Future studies should involve wider samples of participants. This would allow the data to capture a diversity of experiences and viewpoints.

## Conclusion

This work examines the implications of the German frameworks governing TOP, according to those who operate within it: women who seek reproductive healthcare, and professionals in the field. Participants emphasised the importance of accessible TOP after receiving the diagnosis of fetal anomaly. They described, however, how difficult this access is in Germany, and the negative impact it has on women. This includes compromised reproductive autonomy, emotional distress and the pressure of hurried decision-making. The study reveals the gap created when prenatal testing is provided in the absence of guaranteed access to TOP and how this impedes women’s wellbeing, despite it being a declared goal in much policy and regulation. Exploring the issue of TOP, including where due to fetal anomaly, is timely. In a reality in which liberal democracies, such as the US, revoke laws granting women access to TOP and restrict their reproductive autonomy, it is important to examine how access to TOP is understood and experienced in a range of cultural contexts. It is especially timely since in a process of analysing its legal framework, the German government appointed in March 2023 a commission on Reproductive Self-Determination and Reproductive Medicine, covering a range of expertise, including sociology, psychology, health sciences, ethics, and law. The commission released a report summarising a year of work [36], while recommending legalising TOP within 12 weeks gestation. It further recommends that the legislature re-regulate the medical indication, since it lacks clarity due to absent criteria for TOPFA after prenatal diagnosis. This shows that winds of change have begun to blow in Germany. It is evidence of a growing recognition of women’s reproductive struggles and needs and will hopefully help change the general sentiment – which currently involves stigmatisation – towards TOP, including due to fetal anomaly. Future study should follow legislative developments and explore empirically the effects of the changes made. As the World Health Organization asserted, ‘Lack of access to safe, affordable, timely and respectful abortion care, and the stigma associated with abortion, pose risks to women’s physical and mental well-being throughout the life-course’ [57]. Germany must, therefore, not only declare that women’s physical and mental wellbeing is the goal but create the framework to ensure it in practice. It is time to disentangle a discourse that positions reproductive rights and disability rights as being in opposition [2] and critically question existing ideological and historical contingencies [58]. With the current double standard, where the framework enables prenatal testing but impedes TOP, this is a step in the right direction. It should be followed by further steps to ensure women’s access to TOP and thereby their wellbeing.

## Supplementary Information

Supplementary Material 1.

Supplementary Material 2.

## Data Availability

Data are available from the UK Data Archive for researchers who meet the criteria for access to confidential data: Horn, Ruth (2023). Non-invasive Prenatal Testing Study: Comparison England, France, Germany, 2021-2022. [Data Collection]. Colchester, Essex: UK Data Service. 10.5255/UKDA-SN-856508. https://reshare.ukdataservice.ac.uk/856508/.
