# Supplementary material for: Women’s wellbeing as an empty declaration? A qualitative exploration of challenges in accessing termination of pregnancy due to fetal anomaly in Germany
Source: BMC Med Ethics. 2025 Mar 21;26:40. doi: 10.1186/s12910-025-01196-3 (PMC11929346; doi:10.1186/s12910-025-01196-3)
Supplement: Supplementary file 1 — Supplementary Material 1. [file 12910_2025_1196_MOESM1_ESM.docx]

**Topic guide 1: semi-structured interviews with professionals**

The questions will be addressed using semi-structured interviews. This means that the questions will be open to allow the person's point of view to emerge and to be taken in consideration. Through the interviews, we will collect participants’ views, experiences and values in their way of expressing themselves, making criticisms, judging what seems important to them, what are expectations, and what is a cause of concern. During the interview, questions may be formulated as follows, with the aim of supporting the participant's subjectivity and not imposing ways of thinking about NIPT.

This is not intended to be a precise script. The following questions are illustrative of the kind of questions which will be asked at interview.

**QUESTIONS:**

**Women’s antenatal screening pathway and introduction of NIPT to routine testing**

1. How long have you been offering NIPT in your practice?
2. How has the introduction of NIPT into routine antenatal care changed the screening pathway of prenatal women?
3. What is the typical pathway of women referred for NIPT? And what are the different stages in carrying out NIPT?

**Information given and discussion**

1. What do you think is important to communicate to women when offering NIPT? How do you explain the purpose of NIPT?
2. Do you perceive any difficulties in communicating the purpose of NIPT to women, and if so, what are they? How do you try to mitigate them?

**Communication of results and decision-making**

1. Once the test is done, who communicates the results to the women? If this is you, how do you communicate the results?
2. What is your experience of how women make use of the results to make decisions about their pregnancy? Do you perceive any difficulties, or do you feel that the test results help women in the decision-making?
3. Is there anything you think would need to change to support women in making reproductive choices based on the test? And what?

**General views on NIPT**

1. Do you think NIPT differs from other screening tests in any significant way?
2. What do you think are the advantages of NIPT?
3. What do you think are the difficulties or ethical issues regarding NIPT that need to be addressed in the future, in particular regarding its introduction to routine care?
4. What do you think of the current regulatory framework, if there is any?
5. How do you think could these issues be addressed and what would need to change?

**Future developments of NIPT**

1. Do you think NIPT should also be available for other genetic abnormalities apart from those already performed? If so, which ones and why?
2. How do you see the future development of NIPT? Do you think there is scope for its wider use in genomic medicine, and how?
3. Are there any other topics we have not covered but which you would like to talk about and think that they are relevant about NIPT and the development of models of good practice?

**The researchers will pay particular attention to topics such as:**

- Counselling and support given
- Decision-making
- Consent
- Information
- Autonomy of women and couples
- Role of professionals
- Practices associated (or not) with laws, regulations and guidelines
- Advantages/risks associated with NIPT/the way it is implemented
- Value of the test
- Future development of the NIPT and its use in genomic medicine
- Other ethical issues
