# Supplementary material for: Women’s wellbeing as an empty declaration? A qualitative exploration of challenges in accessing termination of pregnancy due to fetal anomaly in Germany
Source: BMC Med Ethics. 2025 Mar 21;26:40. doi: 10.1186/s12910-025-01196-3 (PMC11929346; doi:10.1186/s12910-025-01196-3)
Supplement: Supplementary file 2 — Supplementary Material 2. [file 12910_2025_1196_MOESM2_ESM.docx]

**Topic guide 2: semi-structured interviews with women**

The questions will be addressed using semi-structured interviews. This means that the questions will be open to allow the person's point of view to emerge and to be taken in consideration. Through the interviews, we will collect participants’ views, experiences and values in their way of expressing themselves, making criticisms, judging what seems important to them, what are expectations, and what is a cause of concern. During the interview, questions may be formulated as follows, with the aim of supporting the participant's subjectivity and not imposing ways of thinking about NIPT.

This is not intended to be a precise script. The following questions are illustrative of the kind of questions which will be asked at interview.

**QUESTIONS:**

**Consultation, information and discussion**

1. Can you tell us about your experience with NIPT?
2. Can you describe at what point of pregnancy and why NIPT came up as a possible screening test? Was it your healthcare professional (HCP) who brought it up or yourself?
3. If it was brought up by your HCP, have you heard about the test before or was it the first time you heard of NIPT? What did you think about the possibility to do the test?
4. How did you discuss the test with your HCP, and what information did you receive about the test?
5. Do you remember what conditions your HCP offered to test for, and why? What speciality was/is your HCP? Was/is it a midwife you see, or a consultant, and what kind of consultant?

**Choice**

1. What made you decide to undergo (or not) the test? Was the decision straightforward for you, or did you hesitate? If so, why?
2. What do you think of the information received and the discussion with your HCP? Did it help you in your choice of whether doing the test or not?
3. Did you discuss the decision with anyone else? Another HCP, your partner, family, friends, a charity?
4. From the time the possibility to undergo NIPT was first brought up to the time you did the test (your blood was taken), how much time did you have to think about it?
5. Were you given any leaflets or written information about the test to take home?

**Results**

1. How many days did you have to wait for your results?
2. What were your thoughts while waiting for the results? Did you talk with others during this time, or try to get further information about the test, and where?
3. Did you know what kind of information you could expect from the test? What were your expectations?
4. Who gave you your results, and how (in consultation, on phone)? Did the person communicating the results to you answer all your questions?

**Decision-making**

1. In case the results showed a higher chance of your baby having a genetic condition, how soon after you had the results, did you make a decision about undergoing further tests or about your pregnancy?
2. Was the decision straightforward for you or did you hesitate what the right thing to do would be? If you hesitated, what were your main concerns and questions?
3. Did you feel like you could talk with your HCP about the decision? And did you feel like you got the kind of support you needed?

**Representations of the child and disability**

1. Did your view of the child or your feelings about the pregnancy change following the test? And if so, how?
2. Did you discuss this with anyone?

**General views**

1. What do you think the advantages of NIPT are? What do you think the difficulties of NIPT are, and do you think there are any particular ethical issues regarding the test?
2. What do you think of being able to have the test publicly funded and the conditions to access it?
3. Based on your own experience and reflection, what would you like to change with regard to how NIPT is offered in the public/private sector?
4. Are there any other topics we have not covered but which you would like to talk about and think that they are relevant with regard to NIPT and the development of models of good practice?

**Discussions will cover topics in order to explore:**

- Experiences and expectations
- Counselling and support given/ Information
- Consent
- Decision-making
- Autonomy of women and couples
- Perceptions and concerns of women/couples
- Perceptions of child and disability
- Role of others: professionals, partners, friends, family, charities
- Ethical issues
